# Effect of trilostane treatment on proteinuria secondary to spontaneous hypercortisolism in dogs

**DOI:** 10.1093/jvimsj/aalag189

**Published:** 2026-09-01

**Authors:** Filippo Tagliasacchi, Federico Fracassi, Maddalena Poli, Laura Dalla Tomba, Stefania Golinelli, Antonio Maria Tardo, Francesca Del Baldo

**Affiliations:** Department of Veterinary Medical Science, University of Bologna, Ozzano dell'Emilia, Bologna, Italy; Department of Veterinary Medical Science, University of Bologna, Ozzano dell'Emilia, Bologna, Italy; Department of Veterinary Medical Science, University of Bologna, Ozzano dell'Emilia, Bologna, Italy; Ospedale Veterinario San Francesco, Milan, Italy; Department of Veterinary Medical Science, University of Bologna, Ozzano dell'Emilia, Bologna, Italy; Department of Veterinary Medical Science, University of Bologna, Ozzano dell'Emilia, Bologna, Italy; Department of Veterinary Medical Science, University of Bologna, Ozzano dell'Emilia, Bologna, Italy

**Keywords:** Cushing, dog, efficacy, hypercortisolism, proteinuria, trilostane

## Abstract

**Background:**

Proteinuria is reported in 68%-71% of dogs with spontaneous hypercortisolism (HC). The long-term effect of trilostane on HC-associated proteinuria remains poorly documented.

**Hypothesis/Objectives:**

To evaluate the efficacy of trilostane on proteinuria in dogs with HC and identify potential clinical, anamnestic, and analytical differences between responders and nonresponders.

**Methods:**

Retrospective longitudinal cohort study including dogs with proteinuric HC treated exclusively with trilostane. Clinical history and laboratory data at diagnosis (T0) were retrieved. When available, urine protein-to-creatinine ratio (UPC), disease clinical score (DCS), blood pressure (BP), and daily trilostane dosage (TD) were recorded at 30, 90, 180, and 365 days (T30, T90, T180, and T365) after treatment initiation. Dogs achieving a ≥ 50% UPC reduction or a final UPC < 0.5 were defined as “responders.”

**Results:**

One hundred sixty dogs met inclusion criteria: 89% had pituitary-dependent HC and 10% adrenal-dependent HC. At diagnosis, 64% were proteinuric. A significant UPC reduction over time occurred (*P* = .0005; *F*(4,153.9) = 5.26), with statistically significant decreases at T90 (*P* = .020; difference, 1.093; 95% CI, 0.115-2.071), T180 (*P* = .031; difference, 1.122; 95% CI, 0.064-2.181), and T365 (*P* = .002; difference, 1.599; 95% CI, 0.436-2.761) compared to baseline. At the last follow-up, 76 dogs were evaluable; 48% were responders and had lower median DCS, BP, and TD (*P* = .029, *P* = .025, and *P* = .048, respectively) than nonresponders.

**Conclusions and Clinical Importance:**

Trilostane induces a significant and sustained reduction in proteinuria in some dogs with spontaneous HC, evident from 3 months of treatment. Responders exhibit better control of hypertension and clinical signs despite lower TD. Nonresponders account for 52% of proteinuric dogs.

## Introduction

Naturally occurring Cushing’s syndrome, or hypercortisolism (HC), is a common endocrinopathy in middle-aged and older dogs, characterized by chronic glucocorticoid excess.^1^ Along with systemic hypertension, proteinuria is a well-recognized manifestation of the disease. Initially reported in 44%-46% of untreated cases, more recent studies document an even higher prevalence, affecting 68%-71% of dogs at diagnosis.^2–9^

Nonselective proteinuria, a mix of high- and low-molecular-weight proteins, was originally described in affected dogs, likely due to increased glomerular permeability and impaired tubular reabsorption from protein tubular overload.^4^ However, recent evidence suggests glomerular proteinuria is the more common pattern in dogs with HC.^10^^,^^11^

In a subset of dogs, proteinuria can be reversible after effective treatment of HC. Nevertheless, despite adequate endocrine control, persistent proteinuria occurs in 21%-38% of cases.^2–5^^,^^10^

Trilostane, a competitive inhibitor of 3β-hydroxysteroid dehydrogenase, is the most widely used medical therapy for HC in dogs.^12^ Its efficacy in controlling cortisol production and clinical signs is well established, yet its effect on proteinuria control remains poorly defined. Available evidence regarding trilostane comes from studies primarily designed to investigate survival or broader renal outcomes rather than the evolution of proteinuria. In these studies, different HC treatment strategies were included, with only a subset of dogs receiving trilostane or having subsequent proteinuria data. Heterogeneous findings have been reported, including partial improvement in proteinuria and hypertension, as well as persistent abnormalities, particularly in dogs with long-standing disease or incomplete hormonal suppression.^4^^,^^13^

Notably, although trilostane is the most widely used therapy for HC in dogs, comprehensive studies specifically evaluating its effect on HC-associated proteinuria are currently lacking. The primary objective of this study was to retrospectively assess the effect of trilostane monotherapy in reducing proteinuria in dogs with spontaneous HC. A secondary objective was to identify potential differences in clinical, anamnestic, and laboratory variables between dogs classified as “responders” or “nonresponders” in terms of proteinuria control.

The hypothesis was that trilostane would yield inconsistent outcomes, with a considerable proportion of dogs showing persistent or progressive proteinuria despite appropriate therapy.

## Materials and methods

### Study design

Single-center, retrospective, longitudinal, observational study on client-owned dogs diagnosed with HC and treated with trilostane alone at the Veterinary Teaching Hospital (VTH) of the University of Bologna between April 2004 and August 2024.

Through a VTH database search, complete clinical, anamnestic, and analytical data were collected at diagnosis (T0) for each dog, including signalment (age, breed, sex, neuter status, and body weight [BW]), history, clinical findings, CBC, routine serum biochemistry, urinalysis, and endocrine test results.

Diagnosis of HC was made in accordance with the consensus statement of the American College of Veterinary Internal Medicine (ACVIM). Suspicion of HC was based on a combination of history (eg, polyuria and polydipsia, polyphagia, and dermatological alterations), physical examination findings (eg, alopecia and abdominal enlargement), hematology (eg, lymphopenia, neutrophilia, and thrombocytosis), biochemistry (eg, increased alanine aminotransferase [ALT], alkaline phosphatase [ALP], and γ-glutamyl transferase [γGT]), and urinalysis (eg, low urine specific gravity [USG] and proteinuria).^14^

For diagnostic confirmation, dogs were required to have at least 1 abnormal result on a dynamic endocrine test (low-dose dexamethasone suppression test [LDDSt] or ACTH stimulation test [ACTHst]). When available, the urine cortisol-to-creatinine ratio (UCCR) was used to support the diagnosis but was never used as the sole diagnostic criterion.^15^ Differentiation of pituitary-dependent hypercortisolism (PDH) from adrenal-dependent hypercortisolism (ADH) was based on a combination of abdominal ultrasonography and endogenous ACTH (eACTH) concentrations, often complemented by computed tomography findings. Results of LDDSt were considered supportive when available.^16^

To be classified as proteinuric, a UPC value had to be available at the time of diagnosis, before initiation of treatment. Proteinuria was defined according to the International Renal Interest Society (IRIS) guidelines as a UPC value > 0.5, in the presence of an inactive urinary sediment (ie, absence of hematuria or pyuria).^17^ Dogs were included only if they had been treated exclusively with trilostane for the management of HC.

Dogs were excluded if they had received systemic or topical corticosteroids, tyrosine kinase inhibitors (TKIs), or renin–angiotensin–aldosterone system (RAAS) antagonists at diagnosis or during the follow-up period. Dogs that were azotemic at diagnosis (serum creatinine [sCr] > 1.4 mg/dL, following IRIS guidelines for staging of chronic kidney disease [CKD] in dogs) were also excluded.^17^

Specific time-points in UPC re-evaluation were excluded from statistical analysis if dogs presented evidence of pyuria and hematuria at the urinary sediment examination, indicating a possible post-renal component of proteinuria, secondary to urinary tract inflammation.^18^ The presence of bacteriuria alone, in the absence of hematuria or pyuria was not considered an exclusion criterion for inclusion in statistical analysis. Finally, specific time-points were also excluded if dogs did not receive their trilostane dose the day before reevaluation.

### Data collection

Data on UPC, clinical disease score (assessed via a standardized questionnaire),^1^ systolic blood pressure (BP), sCr and urea, daily trilostane dose, and administration frequency were collected, when available, at 30, 90, 180, and 365 days after starting trilostane (T30, T90, T180, and T365), with a 20% tolerance window around each time point.

Concerning the clinical disease score, the institution adopted a standardized questionnaire aligned with the ALIVE (Agreeing Language in Veterinary Endocrinology) Cushing’s Clinical Score.^1^ The questionnaire consists of 5 variables (drinking, urination, appetite, appearance, and attitude/activity), scored from 0 to 3. The scoring system has a total score ranging from a minimum of 0 to a maximum of 15; a higher score implies greater severity of the HC clinical signs.

Data about clinical disease scores were considered in statistical analysis every time the scoring system was properly reported in clinical records or if, based on retrospective analysis of clinical history, a precise estimate of the same was possible.

The UPC was measured from single spot urine samples collected by cystocentesis or midstream voiding. Concurrent urinalysis was always performed to exclude factors that could influence UPC results (eg, hematuria, pyuria).

Systolic BP was measured by a noninvasive oscillometric device (Suntech 25, Suntech Medical, USA). Measurements were obtained in a quiet environment after at least 10 min of acclimation, using an appropriately sized cuff placed around a limb or the tail base. The mean of 5 consecutive readings was recorded for each assessment. This procedure is in line with the ACVIM consensus statement on systemic hypertension.^19^

The daily trilostane dose (mg/kg/die) was documented at each visit, including any dose adjustment based on clinical response or cortisol testing results and any variation in administration frequency.

### Data analysis

Statistical analysis was performed using MedCalc Statistical Software version 20.218 (MedCalc Software Ltd, Ostend, Belgium) and JMP Student Edition 18.2 (JMP Statistical Discovery LLC, Cary, NC, USA, 2025).

Continuous data were tested for normality using the Shapiro–Wilk test. Normally distributed data were reported as mean ± SD, non-normal data as median and IQR. Variation of UPC after trilostane treatment was analyzed with a linear mixed-effects model, with time (T0, T30, T90, T180, and T365) as a fixed effect and a random intercept for each subject (dog ID) to account for within-subject correlation. The model allowed an assessment of variation of least square means, adjusted for repeated measures and missing data, rather than raw UPC means or medians.

The Tukey’s honestly significant difference (HSD) post-hoc test was applied for multiple comparisons of UPC reduction between time-points.

Concurrent diabetes mellitus (DM) and trilostane administration frequency were included as fixed effects in the linear mixed-effects model to account for potential confounding. Systolic BP was also included as a fixed effect to evaluate its impact on UPC changes over time. At diagnosis, the association between systolic BP and UPC was further assessed using multivariate analysis and Spearman’s rank correlation test.

According to IRIS guidelines for the management of proteinuric dogs, “responders” to trilostane were dogs whose UPC fell below 0.5 or decreased by ≥ 50% from baseline.^20^

Only dogs with a UPC re-evaluation at least 3 months after starting trilostane were classified as “responders” or “nonresponders.” The 3-month threshold for assessing clinically meaningful UPC reduction was based on previous reports.^4^^,^^10^

Given the non-normal distribution of the data, the nonparametric Wilcoxon rank-sum test was used to compare responders and nonresponders at T0 with respect to age, BW, biochemical variables (ALP, ALT, γGT, sCr, urea, albumin, and cholesterol), and endocrine test results (post-ACTH cortisol and cortisol at T8 of the LDDSt), to identify factors associated with a higher likelihood of response to trilostane. Specifically, ALP, ALT, and γGT were evaluated in the clinical context of HC, whereas sCr, urea, albumin, and cholesterol were assessed in relation to HC-associated persistent renal proteinuria.

The Wilcoxon rank-sum test was used to compare BP, clinical score, trilostane dose, and UPC between responders and nonresponders at T0 and last follow-up (T-last). Differences in trilostane dosing frequency were assessed using Pearson’s χ^2^ test.

Dogs were also stratified according to the achievement of proteinuria resolution (UPC < 0.5) and classified as “normalizers” or “non-normalizers.” Comparisons of the same clinical and analytical variables were then performed between the 2 groups.

Finally, to assess whether renal function variables significantly increased during trilostane treatment, the paired Wilcoxon signed-rank test was used to compare T0 and T-last values in the overall study sample and within the responder and nonresponder groups analyzed separately.

## Results

### Study sample

**Figure 1:**
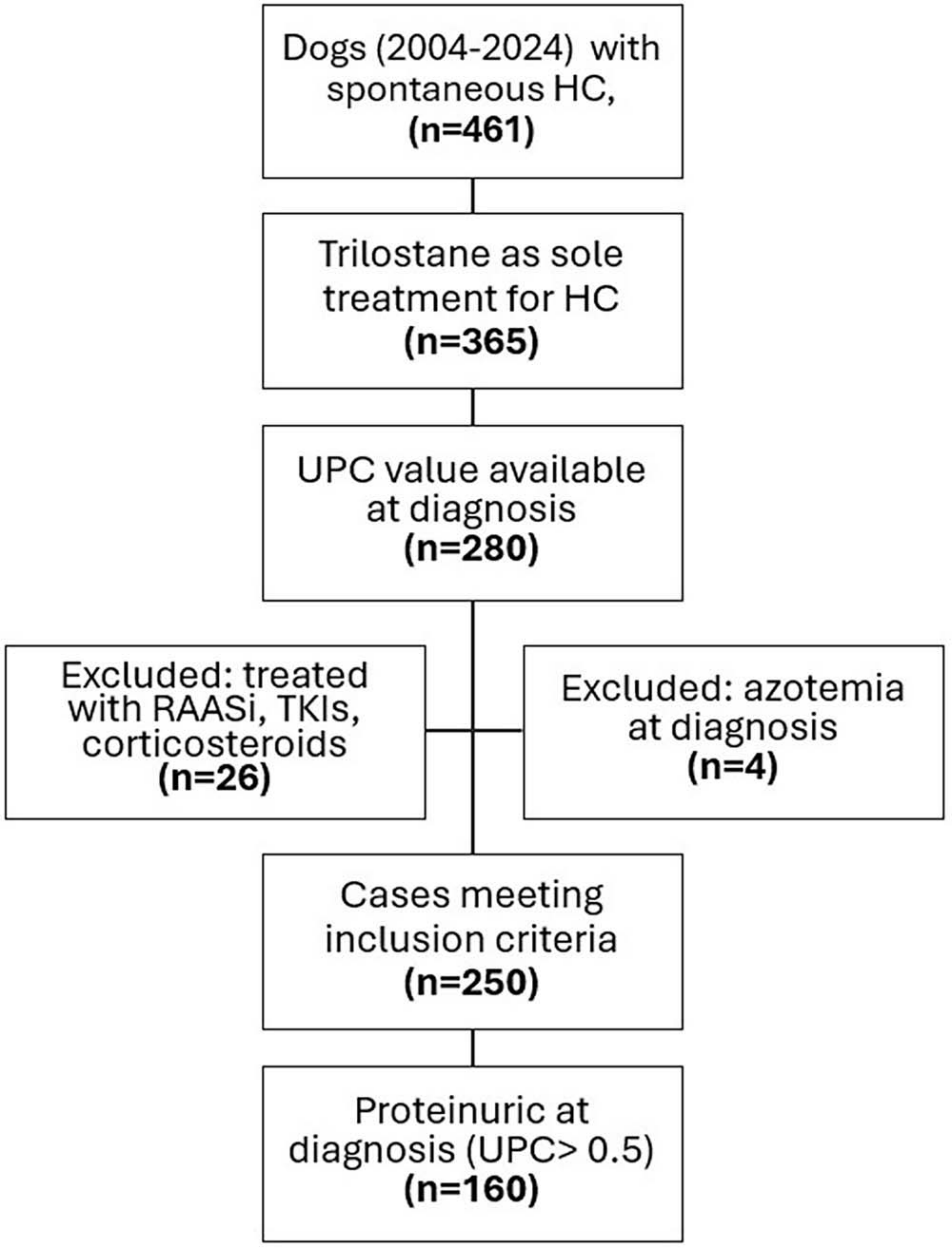
Flow diagram of case enrollment in the study. Abbreviations: HC = hypercortisolism; RAASi = renin–angiotensin–aldosterone system inhibitors; TKIs = tyrosin-kinases inhibitors; UPC = urine protein-to-creatinine ratio.

From April 2004 to August 2024, 461 dogs diagnosed with spontaneous HC at the VTH of the University of Bologna were identified. Trilostane was the sole treatment in 365 dogs, and a UPC value at diagnosis was available for 280. Twenty-six dogs receiving corticosteroids, TKIs, or RAAS inhibitors, and 4 azotemic dogs were excluded. Of the remaining 250 dogs, 64% were proteinuric at diagnosis (Figure 1).

Specifically, out of the 160 proteinuric dogs included, there were 88 males (56 intact, 32 neutered) and 72 females (15 intact, 57 neutered). Median age was 10.6 years (IQR 8.8-12.6). Ninety-two were mixed-breed and 68 purebred; common breeds included Dachshund (*n* = 22), Maltese (*n* = 13), Poodle (*n* = 11), Yorkshire Terrier (*n* = 11), Jack Russell Terrier (*n* = 8), and Boxer (*n* = 6). Endogenous ACTH concentrations were available for 111/160 dogs, while abdominal ultrasonography was performed in 107/160 dogs. Only one dog lacked both eACTH and abdominal ultrasound data; however, total-body CT was available in that case. Based on the available diagnostic results, 142/160 dogs (89%) were diagnosed with PDH, 17/160 (10%) with ADH, and a mixed form was diagnosed in only one dog. Eight dogs had concurrent DM.

### UPC variation across time-points

**Figure 2:**
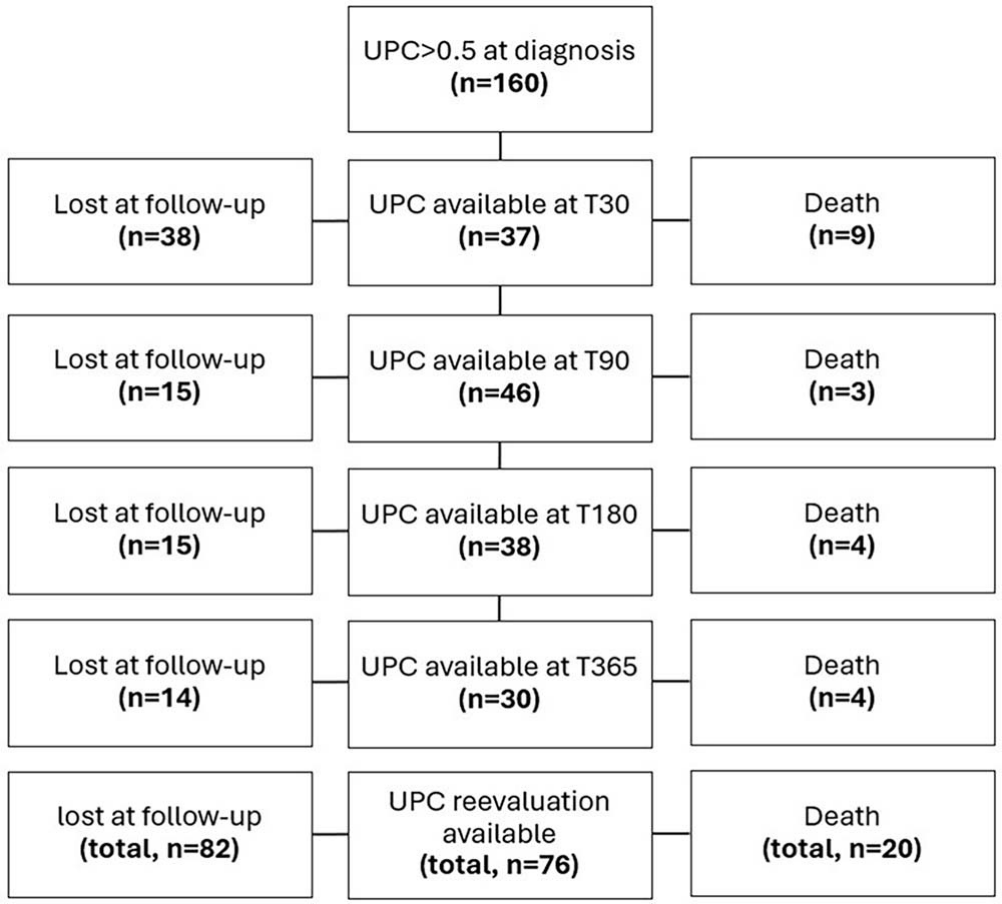
Flow diagram illustrating the sample size and the number of subjects lost to follow-up or deceased at the various timepoints considered. Abbreviation: UPC = urine protein-to-creatinine ratio.

The mixed-effects model showed that the random intercept (dog ID) was significant (*P* = .0001), reflecting variability among individual dogs. Time also had a significant effect (*P* = .0005), indicating that least squares means of UPC differed across time-points even after accounting for repeated measurement and missing data. Details on case numbers at each time-point are reported in Figure 2.

**Figure 3:**
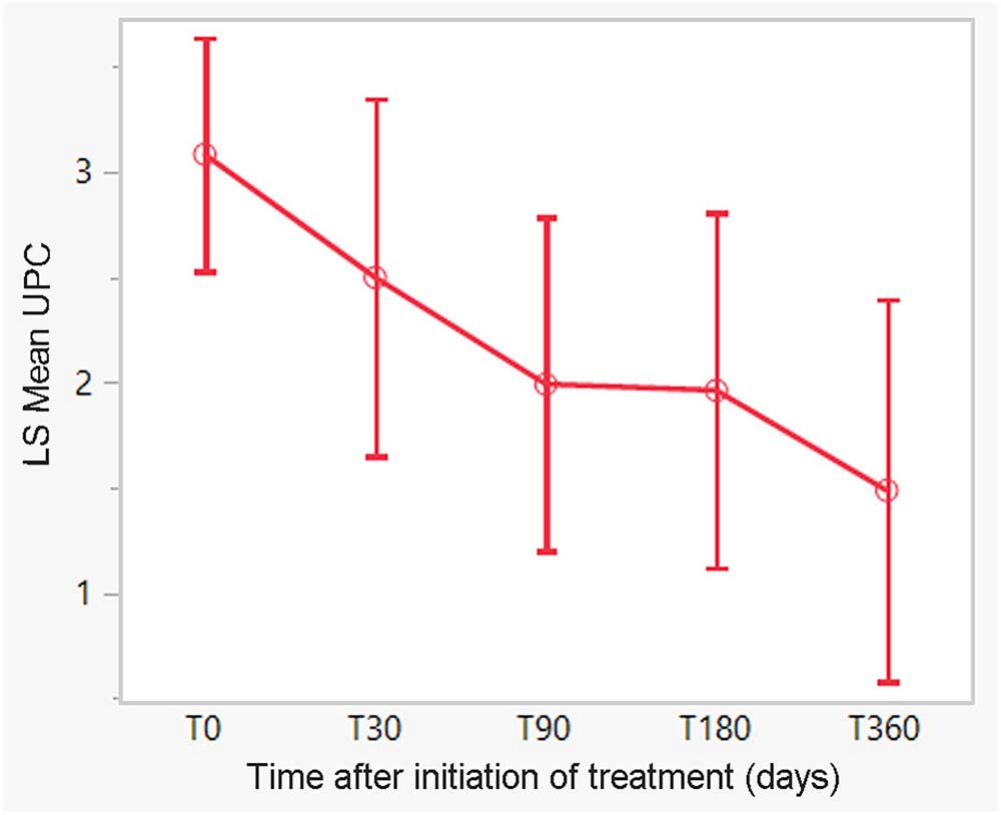
Least-square mean (LSM) UPC values across time points (T0, T30, T90, T180, and T360). Results of the linear mixed-effects model including time as a fixed effect and dog ID as a random intercept to account for repeated measurements with missing data. Data points represent LSMs, and vertical brackets denote 95% CIs. Abbreviations: LS = least square; UPC = urine protein-to-creatinine ratio.

Least-squares means of UPC across time-points are graphically reported in Figure 3. The model-adjusted estimates, accounting for repeated measures and missing data, show a trend in decreasing already at T30. However, a statistically significant reduction occurs at T90 (*P* = .020), as confirmed by the Tukey’s HSD test for post-hoc multiple comparison between time-points. The statistically significant reduction compared to baseline is maintained at T180 (*P* = .031) and T365 (*P* = .002). Details on point-estimates and measures of uncertainty on the post-hoc test are reported in Table 1.

**Table 1:** Multiple comparisons of least-squares mean of urine protein-to-creatinine ratio between timepoints through Tukey’s HSD post-hoc test.

Time point vs	Time point	Difference	Prob > |*t*|	<95%	>95%
**T0**	**T30**	0.585	.558	−0.485	1.656
**T0**	**T90**	1.093	.020^*^	0.15	2.071
**T0**	**T180**	1.122	.031^*^	0.064	2.181
**T0**	**T360**	1.599	.002^*^	0.436	2.761

Abbreviations: HSD, honestly significant difference; Prob, probability; < 95%, inferior limit of 95% CI; > 95%, superior limit of 95% CI. ^*^Indicates statistical significance (*P* < .05).

Point-estimates and measures of uncertainty are expressed through the difference between least-squares means and the 95% CI.

According to the mixed-effects model, the presence of concurrent DM did not show a significant effect on UPC variation across time-points, with no significant association (*P* = .42).

### Systolic blood pressure and UPC variation

The multivariate analysis and Spearman’s rank correlation test indicated that systolic BP was not significantly associated with the degree of proteinuria at time of diagnosis (*P* = .28). Moreover, according to the mixed-effects model, systolic BP did not show a significant effect on UPC variation across time-points, with no significant association (*P* = .89).

### Responders vs nonresponders

A UPC value was available at 1 follow-up after starting trilostane in 76 of 160 dogs. In 67 of these, a UPC value was available after at least 3 months of therapy.

At the last follow-up, 47.8% (32/67) of dogs were defined as responders to trilostane in terms of UPC reduction. At T0, no significant differences in age, UPC, disease clinical score, systolic BP, or trilostane daily dosage were found between responders and nonresponders. The only significant clinical difference was BW (*P* = .038), with responders having higher median BW (12.7 kg [7.4-17.8]) than nonresponders (9.3 kg [7.4-12.7]). Details are provided in Table 2. No differences were observed in serum biochemistry (ALP, ALT, GGT, albumin, and cholesterol) or endocrine tests (poststimulation cortisol at ACTHst and T8 cortisol at LDDSt; Table 2).

**Table 2:** Comparison between “responders” and “nonresponders” to trilostane based on clinical and analytical variables (serum biochemistry and endocrine test results) at the time of diagnosis through Wilcoxon rank-sum test.

Variables T0 (diagnosis)	Responders—median (IQR I-III)	*N*	Nonresponders—median (IQR I-III)	*N*	*P* value
**Age (years)**	10.3 (7.8-12.3)	30	10.6 (9-12.1)	34	.78
**Weight (kg)**	12.7 (7.4-17.8)	31	9.3 (7.4-12.7)	33	.038^*^
**Clinical score**	6 (4-8)	31	6 (3-7)	35	.31
**NIBP (mmHg)**	152 (134.5-173)	13	144 (133-160)	19	.27
**UPC**	1.95 (1.03-5.42)	31	2.1 (0.92-3.74)	35	.86
**Trilostane (mg/kg/day)**	1.54 (1.33-1.92)	11	1.96 (1.2-2.56)	21	.54
**ALP (U/L)**	873 (587-3170.2)	31	689.5 (353-2357.2)	34	.45
**ALT (U/L)**	190 (109-403)	31	162 (83.7-306)	34	.43
**γGT (U/L)**	21.7 (7.9-83.2)	29	22 (8.9-48.25)	33	.74
**CHOL (mg/dL)**	424 (304.5-546.5)	29	432 (336.5-532)	34	.60
**ALB (g/dL)**	3.06 (2.81-3.28)	30	3.19 (2.89-3.45)	34	.31
**LDDST8 (μg/dL)**	3.54 (1.94-9.15)	14	2.7 (1.85-4.98)	17	.31
**Post-ACTH cortisol (μg/dL)**	33.3 (21.2-44.7)	27	36.6 (26.6-45.4)	27	.21

Abbreviations: ALB, albumin; ALP, alkaline phosphatase; ALT, alanine aminotransferase; CHOL, cholesterol; ALB, albumin; γGT, γ-glutamyl transferase; LDDST8, T8 cortisol concentration at the low-dose dexamethasone suppression test; *N*, numerosity; NIBP, noninvasive blood pressure; post-ACTH cortisol, post-ACTH administration cortisol concentration; UPC, urine protein-to-creatinine ratio. Bold values and ^*^indicate statistically significant results (*P* < .05).

In the “*N*” column is reported the total number of dogs on which the data are calculated.

**Figure 4:**
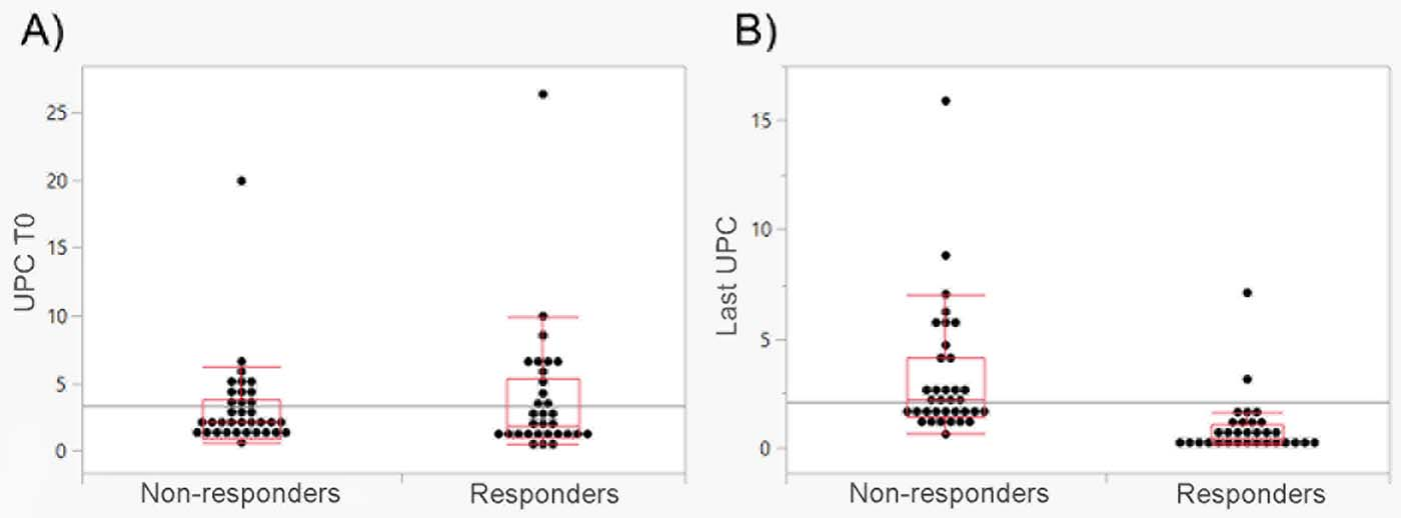
(A) Comparison of UPC between responders and nonresponders to trilostane treatment at the time of diagnosis (T0). Each dot represents an individual patient’s result. The bar represents the median, and the whiskers represent the IQR. *P* = .86. (B) Comparison of UPC between responders and nonresponders to trilostane at the last available follow-up. Each dot represents an individual patient’s result. The bar represents the median, and the whiskers represent the IQR. *P* < .0001. Abbreviation: UPC = urine protein-to-creatinine ratio.

At the last follow-up, responders had lower median UPC than nonresponders (0.42 [0.2-1.11] vs 2.2 [0.92-3.74], *P* < .0001). Median UPC in nonresponders remained stable from baseline (Figure 4).

**Figure 5:**
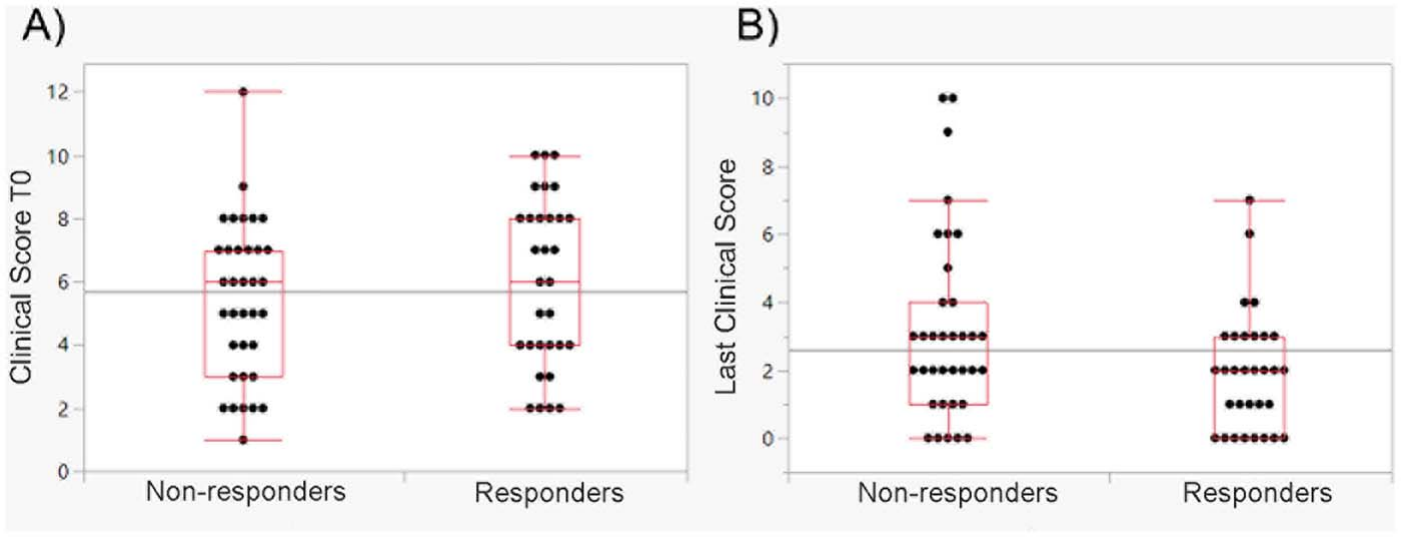
(A) Comparison of clinical disease score between responders and nonresponders to trilostane at the time of diagnosis (T0). Each dot represents an individual patient’s result. The bar represents the median, and the whiskers represent the IQR. *P* = .31. (B) Comparison of clinical disease score ratio between responders and nonresponders to trilostane at the last available follow-up. Each dot represents an individual patient’s result. The bar represents the median, and the whiskers represent the IQR. *P* = .029.

**Figure 6:**
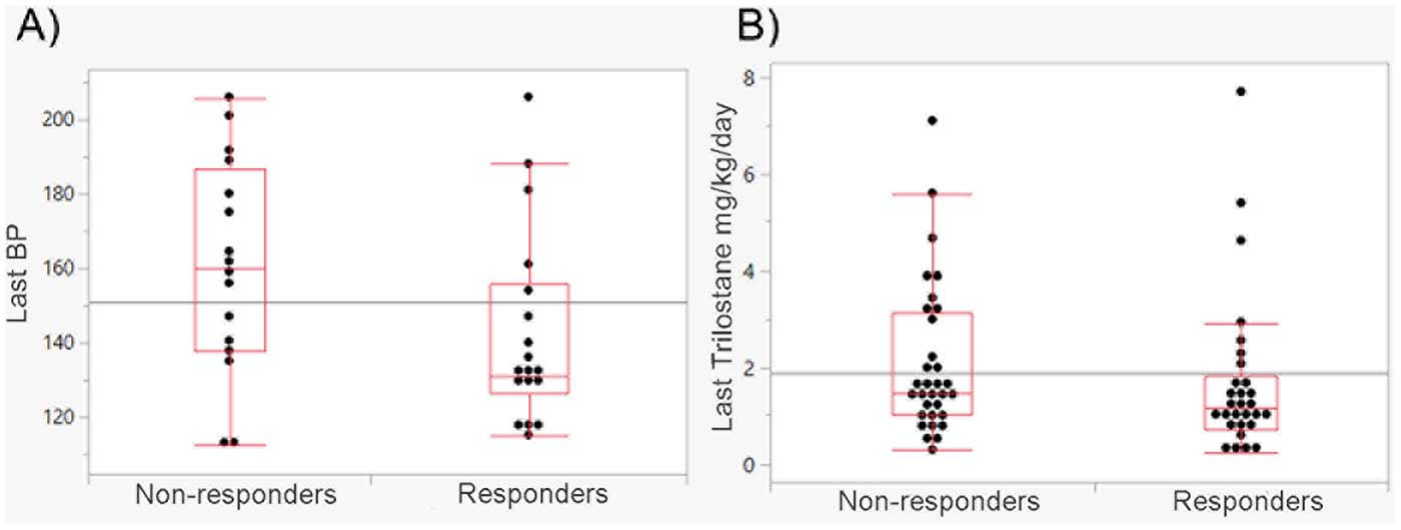
(A) Comparison of BP between responders and nonresponders to trilostane at the last available follow-up. Each dot represents an individual patient’s result. The bar represents the median, and the whiskers represent the IQR. *P* = .025. (B) Comparison of trilostane daily dosage between responders and nonresponders to trilostane at the last available follow-up. Each dot represents an individual patient’s result. The bar represents the median, and the whiskers represent the IQR. *P* = .048. Abbreviation: BP, blood pressure.

For disease clinical score, both groups showed median reductions at T-last compared to baseline, but responders had lower scores than nonresponders (2 [0-3] vs 3 [1-4]; *P* = .029; Figure 5). Responders also had lower BP (131 mmHg [126.3-155.8] vs 160 mmHg [137.8-187.8]; *P* = 0.025) and lower trilostane daily dosage (1.15 mg/kg/d [0.72-1.84] vs 1.46 mg/kg/d [1-3.1]; *P* = 0.048; Figure 6). At T30, 50% of dogs had a trilostane dose adjustment. Details about the comparisons at T-last are reported in Table 3.

**Table 3:** Comparison between “responders” and “nonresponders” to trilostane based on variables (urine protein-to-creatinine ratio, clinical disease score, blood pressure, and trilostane daily dosage) at the last available follow-up through Wilcoxon rank-sum test.

Variables (last follow-up)	Responders—median (IQR I-III)	*N*	Nonresponders—median (IQR I-III)	*N*	*P* value
**Clinical score**	2 (0-3)	31	3 (1-4)	35	.029^*^
**NIBP (mmHg)**	131 (126.2-155.7)	18	161 (137.7-186.7)	16	.025^*^
**UPC**	0.42 (0.2-1.1)	31	2.2 (1.4-4.1)	35	<.0001^*^
**Trilostane (mg/kg/day)**	1.15 (0.72-1.84)	29	1.46 (1-3.15)	32	.041^*^

Abbreviations: *N*, numerosity; NIBP, noninvasive blood pressure; UPC, urine protein-to-creatinine ratio. ^*^Indicates statistical significance (*P* < .05).

In the “*N*” column is reported the total number of dogs on which the data are calculated.

### Normalizers vs non-normalizers

At the last-available follow-up, a UPC reduction below 0.5 was verified in only 19 out of 67 dogs (28.4%), classified as “normalizers” according to previous definitions. At T0, no statistically significant differences were found between the normalizers and non-normalizers in terms of age, BW, clinical disease score, systolic BP, or trilostane daily dosage. The only variable differing at diagnosis was median UPC, being significantly lower in normalizers (1.13 [0.7-2.4]) compared with non-normalizers (2.01 [1.36-4.91]). Details of the comparisons at T0 are reported in Table SS1. No significant differences were observed in ALP, ALT, GGT, albumin, cholesterol, or endocrine test results (post-ACTH cortisol and T8 cortisol at LDDSt; Table SS1).

At T-last, normalizers had significantly lower UPC than non-normalizers (0.225 [0.15-0.37] vs 1.56 [1.22-3]; *P* < .0001). Both groups showed reduced clinical scores from baseline, but values remained lower in normalizers (1.5 [0-3] vs 2 [1-3.75]; *P* = .039). Normalizers also had lower systolic BP (130 mmHg [117-131] vs 159 mmHg [140-188]; *P* = .0015) and trilostane dose (1 mg/kg/day [0.83-1.31] vs 1.46 mg/kg/day [0.92-2.98]; *P* = .027). Details are reported in Table SS2.

### Frequency of trilostane administration

Among dogs included in the responder vs nonresponder analysis, trilostane dosing frequency was available for 50/67 subjects at diagnosis and 39/67 at ≥ 3 months follow-up. Administration frequency was not associated with response status at diagnosis (Pearson’s χ^2^ test; *P* = .74) or at T-last (Pearson’s χ^2^ test; *P* = .77). In the mixed-effects model, dosing frequency had no significant effect on UPC variation over time (*P* = .58).

### Renal variables

Among dogs included in the responder vs nonresponder comparison, sCr data were available at diagnosis and ≥ 3 months follow-up for 66/67 subjects, and urea for 55/67. At diagnosis, no differences in sCr or urea were observed between responders and nonresponders (*P* = .20 and *P* = .66, respectively). At follow-up, responders had higher sCr than nonresponders (1.01 mg/dL [0.69-1.14] vs 0.79 mg/dL [0.60-1.01], *P* = .026), whereas no difference in urea was detected (*P* = .59).

When comparing T0 vs T-last, in the overall study sample both sCr (0.76 mg/dL [0.64-0.91] vs 0.83 mg/dL [0.64-1.06]; *P* = .04) and urea (30 mg/dL [19-38] vs 45 mg/dL [28-63]; *P* = .0045) increased. In responders, both sCr (0.76 mg/dL [0.63-0.90] vs 1.01 mg/dL [0.68-1.14]; *P* = .023) and urea (29 mg/dL [21.3-41] vs 38 mg/dL [26-64]; *P* = .047) increased. In nonresponders, sCr did not change (*P* = .76), whereas urea increased (30 mg/dL [28-63] vs 45 mg/dL [28-63]; *P* = .0045).

## Discussion

In the present study, treatment with trilostane resulted in a significant and sustained reduction in proteinuria secondary to spontaneous HC in dogs, with the effect becoming significant from 3 months after initiation of therapy. Only 48% of dogs achieved the therapeutic goal for proteinuria reduction with trilostane, while complete resolution occurred in 28% of dogs. Moreover, the group of dogs classified as “responders” to trilostane showed better management of HC-associated abnormalities, including typical clinical signs (polyuria, polydipsia, polyphagia, and dermatological alterations) and systemic hypertension.

The delayed onset of the antiproteinuric effect is consistent with that previously reported.^4^ In the subgroup of 13 dogs treated exclusively with trilostane, a significant decrease in median UPC values occurred 3 months after starting medical therapy. Several hypotheses could explain this delayed response: first, the time required during medical treatment with trilostane to achieve adequate hypercortisolism control, and second, the need to reverse vascular, renal, and hemodynamic changes that develop secondary to this chronic process.

From a clinical standpoint, in dogs diagnosed with HC and treated solely with trilostane, the first clinical and hormonal re-evaluation typically occurs about 4 weeks after initiating therapy, unless early adverse effects arise.^15^ Achieving adequate HC control could therefore require dosage adjustments over several weeks.^12^ Several weeks are usually needed to reduce polyuria, polydipsia, and polyphagia, while several months might be required before visible improvement in dermatological lesions is observed.^15^

This adjustment period, primarily driven by the identification of an effective therapeutic dose, could reflect the delayed improvement in proteinuria control. Supporting this hypothesis, in the study sample 50% of dogs required dosage adjustments 30 days after starting trilostane, based on clinical and laboratory findings, indicating suboptimal disease control. It is therefore likely that the significant reduction in proteinuria observed after the third month of therapy reflects both improved disease control in the recruited cohort and the gradual resolution of functional hemodynamic stress associated with HC.

Consistent with previous studies, 64% of dogs in the present study sample were proteinuric at diagnosis.^4^^,^^5^^,^^11^ This proportion is higher than what has been reported in some other studies.^2^^,^^3^ Differences in diagnostic thresholds used to define proteinuria (UPC > 1.0 vs > 0.5) and in the methodologies for quantifying urinary proteins could partially account for this discrepancy.^4^^,^^11^^,^^21^^,^^22^

The pathogenesis of proteinuria in HC is multifactorial and not yet fully elucidated. Excess glucocorticoids induce glomerular hyperfiltration, endothelial dysfunction, and greater glomerular basement membrane (GBM) permeability.^3^^,^^5^ The resulting proteinuria is generally glomerular or nonselective in nature, reflecting a combined glomerular and tubular origin.^4^^,^^11^^,^^23^ Recent evidence indicates that glomerular proteinuria represents the predominant pattern in dogs with spontaneous HC.^10^^,^^11^

Additionally, chronic cortisol-mediated activation of the RAAS, sodium retention, and increased vascular sensitivity to catecholamines promote the development of systemic hypertension, affecting approximately 50%-75% of dogs with HC.^6^^,^^12^ Overall, hypertension and proteinuria establish a cycle of glomerular stress, through mechanisms analogous to those described in human Cushing’s syndrome.^24^^,^^25^

Given the complexity of the underlying pathophysiological mechanisms, it is not surprising that only 48% of dogs in the present study achieved the therapeutic goal for proteinuria reduction with trilostane. This proportion is slightly lower than in earlier studies,^3^^,^^4^ but aligns with more recent findings.^10^ A relevant factor is that earlier studies also included dogs treated surgically (adrenalectomy, hypophysectomy), with radiotherapy, or with mitotane (o,p′-DDD).^3^^,^^4^

When considering only dogs treated with trilostane in a previous study, 43% did not achieve UPC normalization.^4^ Similarly, in a recent study, more than half of the 7 dogs with available follow-up data after treatment initiation showed inadequate response to trilostane. In the same study, only 2 of 7 dogs (28.5%) reached a UPC below 0.5.^11^ These findings are fully consistent with the results of the larger cohort in the present study.

The lack of response to trilostane in more than half of the dogs could stem from several factors. First, the mechanism of action: trilostane, a synthetic steroid analogue, acts by competitively inhibiting the steroidogenic enzyme 3-β-hydroxysteroid dehydrogenase, ultimately blocking cortisol synthesis. Because it does not address the underlying cause of hypercortisolism, treatment can be associated with daily fluctuations in cortisol concentrations, sometimes exceeding the reference range. “Temporary hypercortisolemia” could occur even in dogs with apparently adequate clinical control of HC. Although transient, HC could maintain a state of hypertension and glomerular hyperfiltration, delaying measurable improvement in proteinuria.^4^

Although temporary hypercortisolemia is a plausible hypothesis, no differences were observed between responders and nonresponders based on trilostane administration frequency. Administration frequency was also not significantly associated with UPC variation over time in the mixed-effects model. A greater impact of temporary hypercortisolemia might be expected in dogs receiving trilostane once daily, as its activity can be equal to or shorter than 12 h in some individuals.^26^

Persistent proteinuria in nonresponders could also reflect irreversible outcomes of chronic glomerular remodeling, endothelial dysfunction, and tubulointerstitial fibrosis secondary to prolonged glucocorticoid excess. Histopathological studies have documented segmental glomerulosclerosis, mesangial expansion, GBM thickening, and interstitial fibrosis in dogs with HC.^3^^,^^27^^,^^28^ These changes could contribute, wholly or partially, to persistent proteinuria despite apparent endocrine remission.

In our sample, no significant differences were identified between responders and nonresponders at diagnosis in terms of age, clinical disease score, UPC, BP, or trilostane dose. The only clinical variable that differed significantly between responders and nonresponders at diagnosis was BW, with responders showing higher weights. This finding could reflect characteristics specific to the study sample rather than a broadly applicable association. The only supporting evidence in the literature comes from a retrospective study of 70 dogs with PDH, which reported that dogs weighing > 30 kg could require lower trilostane doses per kilogram to achieve clinical control. Although no significant differences were observed across other weight categories, regression analysis suggested a trend toward decreasing total daily dosage in dogs weighing > 15 kg.^29^

At the last available follow-up, responders showed a significantly lower clinical score and lower systolic BP, although the latter must be interpreted cautiously due to the limited number of available measurements. Notably, responders received a significantly lower median trilostane dose than nonresponders at the end of follow-up.

Results regarding clinical scores and BP support the hypothesis that medical reversal of HC through trilostane could promote normalization of hemodynamic, vascular, and renal abnormalities secondary to glucocorticoid excess. The lower trilostane dose in responders can depend on various factors: in clinical practice, dose escalation is determined by both hormonal test results and persistence or resolution of clinical signs attributable to HC.^15^ It is therefore plausible that, in poorly responsive dogs, persistent clinical signs prompted progressive dose increases, resulting in a higher median value in nonresponders. Failure to respond to escalating dosages of trilostane is currently one of the criteria prompting consideration of alternative therapeutic options or further investigation of HC etiology.

At diagnosis, 82% of dogs in the present study were hypertensive, consistent with literature.^6^^,^^12^ Responders showed a lower final BP than nonresponders, and the prevalence of hypertension among responders at the last available follow-up was 37%, compared with 74% in nonresponders. This finding suggests that dogs with persistent proteinuria tended to have higher systolic BP during follow-up; however, consistent with previous studies, BP was not significantly associated with UPC at diagnosis or during follow-up.^2^^,^^4^ The underlying pathophysiological mechanism of proteinuria in dogs affected by HC is complex and multifactorial. In the present study, not only did the severity of proteinuria at diagnosis fail to correlate with BP, but changes in UPC during trilostane treatment also did not appear to be a direct consequence of BP control. Despite this, dogs classified as nonresponders to trilostane treatment at the end of follow-up, showed a significantly increased median BP compared to responders.

Overall, the relationship between BP and proteinuria in this cohort should be interpreted as associative rather than causative. Systemic BP could represent one of several factors involved in the development of proteinuria in dogs with HC. In nonresponders, the presence of a significantly higher median systolic BP compared with responders could be the result of chronic activation of the RAAS secondary to proteinuria itself.

Whether persistent renal proteinuria and hypertension are negative prognostic indicators in HC remains to be determined in veterinary medicine concerning the dog. Persistent proteinuria is also rarely reported in human HC, and evidence remains inconclusive. Assessing the impact of persistent renal proteinuria on outcomes was not an objective of the present study. Descriptively, at the last available follow-up, creatinine values were available for 55 dogs. Five dogs were azotemic: 2 had borderline values, 2 dogs met criteria for classification in IRIS-CKD 2 stage, and only 1 was classified as belonging to IRIS-CKD 3 stage.^20^ All azotemic dogs had a UPC > 0.5 at the last measurement, but 2 of the 5 were classified as responders based on a ≥ 50% reduction in UPC from baseline. Although median concentrations remained within the reference interval, sCr and urea significantly increased from T0 to T-last in the overall study sample. Among responders, both variables increased over time, whereas in nonresponders only urea increased, with sCr remaining unchanged. These results are consistent with a previous prospective study, which reported increased glomerular filtration rate and subsequent posttreatment rises in sCr in dogs with ACTH-dependent HC, although values remained within the reference interval.^4^ It was not possible to determine whether azotemia developed secondary to HC-related hypertension and proteinuria or resulted from progression of pre-existing IRIS-CKD stage 1. Previous studies, based on tubular and glomerular biomarkers, have found evidence of both glomerular and tubular dysfunction in untreated Cushing’s syndrome in dogs.^4^^,^^30^ Pituitary-dependent HC can be associated with hyperfiltration and altered mineral homeostasis with increased fibroblast growth factor 23 (FGF23) concentrations, reinforcing the concept of combined glomerular and tubular dysfunction.^23^^,^^31^ In the long-term, in humans, decreased glomerular filtration rate occurs.^25^

Persistent renal proteinuria and hypertension are recognized as risk factors for progression and mortality in dogs with CKD.^32–35^ In terms of signalment and clinical signs, similarities exist between dogs with CKD and those with HC: both diseases primarily affect adult to geriatric dogs, and both frequently present with polyuria, polydipsia, hypertension, and predisposition to urinary tract bacterial colonization. Dogs with Cushing’s syndrome could therefore be at risk of developing renal complications.^36^ Given these shared clinical features, it is reasonable to hypothesize that persistent renal proteinuria could represent a therapeutic target in HC management.

Prospective studies with larger cohorts are needed to determine whether persistent renal proteinuria in HC predisposes to CKD, or increased mortality. Despite being unsupported by specific evidence, the use of RAAS inhibitors for persistent proteinuria in HC dogs treated with trilostane, in accordance with proteinuria management guidelines, should be considered. However, caution is warranted because of potential adverse effects, particularly hyperkalemia. Adjunctive nutritional strategies (eg, moderate sodium restriction and management of hyperlipidemia or hyperphosphatemia) could also support renal and vascular protection.

The main limitations of this study arise from its retrospective design. Missing UPC and BP data occurred at several time points for most dogs. The main causes of data unavailability were loss to follow-up (51.2%) and death (12.5%) before T365. These factors reduced the number of evaluable cases, potentially introducing selection bias and affecting observed response rates. The high proportion of lost cases could reflect the referral nature of the caseload, with some dogs returning to their primary veterinarians for follow-up.

In both groups (responders and nonresponders), a subset of dogs showed persistently high clinical scores at the last follow-up, suggesting incomplete HC control. In nonresponders, a proportion of these cases could reflect the 10%-15% of dogs with HC previously described as refractory to medical treatment with trilostane.^37^ Nonetheless, in both groups, further dose adjustments might have led to improved endocrine control and therefore possibly better proteinuria outcomes at subsequent follow-ups. Furthermore, the retrospective reconstruction of clinical scores from medical records in some time-points could have introduced subjective bias.

Given the advanced mean age of the study sample, concurrent diseases such as early-stage CKD cannot be excluded in all dogs. Eight of 160 dogs had concurrent, clinically controlled DM at inclusion, 6 of which were included in the trilostane response analysis. As 20%-55% of diabetic dogs can exhibit UPC above the reference range, DM could theoretically have contributed to the persistently increased UPC in this subgroup.^38^^,^^39^ Accordingly, all 6 diabetic dogs were classified as nonresponders. However, when DM was included as a fixed effect in the mixed-effects model, it did not substantially influence the longitudinal UPC pattern in this cohort.

Additionally, inclusion was limited to proteinuric dogs at diagnosis, preventing assessment of proteinuria development during treatment.

The 20-year retrospective recruitment period is a study limitation, as diagnostic approaches, HC management, and laboratory methods have changed over time. Different assays for serum cortisol and UPC could have been used, which should be considered when interpreting results. Additionally, UPC was measured from single spot urine samples, reflecting routine practice but not accounting for known intra-individual variability, as repeated measurements were not performed.^40^

Similarly, the intrinsic variability of noninvasive BP measurement methods represents a potential source of bias. Finally, data on dietary management were not available, and certainly not standardized.

## Conclusion

Trilostane therapy in dogs with HC significantly reduces proteinuria over time, with improvements detectable after ~ 3 months, though complete UPC normalization is uncommon. Responders show lower systolic BP, greater clinical improvement, and lower daily trilostane doses, suggesting that sustained hormonal control and vascular adaptation are key for renal outcomes. Persistent proteinuria and hypertension likely reflect irreversible glomerular changes, highlighting the need for prospective studies on adjunctive antiproteinuric and antihypertensive strategies. Overall, these findings reinforce that cortisol excess is a systemic disorder with renal and cardiovascular effects beyond endocrine dysregulation, emphasizing the value of an integrated therapeutic approach in veterinary medicine.

## Supplementary Material

Supplementary_material_aalag189

## Off-label antimicrobial declaration

The authors declare no off-label use of antimicrobials.

## Institutional animal care and use committee or other approval declaration

As all dogs included in this study were presented for routine clinical rechecks, which are part of the standard care protocol for all dogs with hypercortisolism at our institutions, no additional stress or procedures were imposed on the animals to collect the data used in this study. For this reason, ethical approval was not required.

## Abbreviations

ACTHst: ACTH stimulation test
ACVIM: American College of Veterinary Internal Medicine
ADH: adrenal-dependent hypercortisolism
ALP: alkaline phosphatase
ALT: alanine aminotransferase
BP: blood pressure
CKD: chronic kidney disease
eACTH: endogenous ACTH
GBM: glomerular basement membrane
γGT: γ-glutamyl transferase
HC: hypercortisolism
IRIS: International Renal Interest Society
LDDSt: low-dose dexamethasone suppression test
PDH: pituitary-dependent hypercortisolism
RAAS: renin–angiotensin–aldosterone system
TKIs: tyrosine kinase inhibitors
UCCR: urine cortisol-to-creatinine ratio
UPC: urine protein-to-creatinine ratio
USG: urine specific gravity
VTH: Veterinary Teaching Hospital
