# Supplementary material for: Effect of trilostane treatment on proteinuria secondary to spontaneous hypercortisolism in dogs
Source: J Vet Intern Med. 2026 Sep 1;40(5):aalag189. doi: 10.1093/jvimsj/aalag189 (PMC13533187; doi:10.1093/jvimsj/aalag189)
Supplement: Supplementary file 1 [file supplementary_material_aalag189.zip › Supplementary_table_1_aalag189.docx]

| **Variables T0 (diagnosis)** | **Normalizers - median (IQR I-III)** | **N** | **Non-normalizers - median (IQR I-III)** | **N** | **P value** |
| --- | --- | --- | --- | --- | --- |
| **Age (years)** | 10.3 (8.2-12.0) | 19 | 10.6 (8-12.5) | 46 | 0.96 |
| **Weight (kg)** | 13.35 (7.2-20.7) | 18 | 9.5 (7.45-15) | 46 | 0.17 |
| **Clinical score** | 6.5 (4-8.25) | 19 | 6 (3.25-7) | 48 | 0.29 |
| **NIBP (mmHg)** | 144 (126.2-151.5) | 8 | 150 (137.2-169.2) | 24 | 0.27 |
| **UPC** | 1.13 (0.7-2.4) | 19 | 2.26 (1.36-4.91) | 48 | **0.0041*** |
| **Trilostane (mg/kg/die)** | 1.62 (1.38-1.99) | 8 | 1.79 (1.2-2.44) | 24 | 0.81 |
| **ALP (U/L)** | 872.5 (527.2-2056) | 19 | 795.5 (378-2277) | 46 | 0.83 |
| **ALT (U/L)** | 178.5 (109-416.5) | 18 | 164 (97-329) | 47 | 0.57 |
| **γGT (U/L)** | 28.8 (7.6-64.7) | 17 | 21.9 (8.15-55.25) | 45 | 0.98 |
| **CHOL (mg/dL)** | 350 (281.5-533) | 17 | 433.5 (346-558) | 46 | 0.17 |
| **ALB (g/dL)** | 3.13 (2.98-3.36) | 17 | 3.1 (2.76-3.41) | 47 | 0.53 |
| **LDDST8 (μg/dL)** | 3.6 (2.21-17.25) | 9 | 2.75 (1.71-5.4) | 22 | 0.13 |
| **Post-ACTH cortisol (μg/dL)** | 30.9 (19.5-44.7) | 15 | 36.6 (26.6-45.4) | 39 | 0.20 |
