# Supplementary material for: Effect of trilostane treatment on proteinuria secondary to spontaneous hypercortisolism in dogs
Source: J Vet Intern Med. 2026 Sep 1;40(5):aalag189. doi: 10.1093/jvimsj/aalag189 (PMC13533187; doi:10.1093/jvimsj/aalag189)
Supplement: Supplementary file 1 [file supplementary_material_aalag189.zip › Supplementary_table_2_aalag189.docx]

| **Variables (Last follow-up)** | **Normalizers - median (IQR I-III)** | **N** | **Non-normalizers - median (IQR I-III)** | **N** | **P value** |
| --- | --- | --- | --- | --- | --- |
| **Clinical score** | 1.5 (0-3) | 19 | 2 (1-3.75) | 48 | **0.039*** |
| **BP (mmHg)** | 130 (117-131) | 11 | 159 (140-188) | 23 | **0.0015*** |
| **Trilostane daily dosage** | 1 (0.83-1.31) | 17 | 1.46 (0.92-2.98) | 44 | **0.027*** |
| **UPC** | 0.225 (0.15-0.37) | 19 | 1.56 (1.22-3) | 48 | **<.0001*** |
