# Supplementary material for: Effect of trilostane treatment on proteinuria secondary to spontaneous hypercortisolism in dogs
Source: J Vet Intern Med. 2026 Sep 1;40(5):aalag189. doi: 10.1093/jvimsj/aalag189 (PMC13533187; doi:10.1093/jvimsj/aalag189)
Supplement: Supplementary file 1 [file supplementary_material_aalag189.zip › Supplementary_Table_caption_aalag189.docx]

**Supplementary Table 1: Comparison between “normalizers” and “non-normalizers” to trilostane based on clinical and analytical variables (serum biochemistry and endocrine test results) at the time of diagnosis through Wilcoxon rank-sum test.** In the “N” column is reported the total number of dogs on which the data are calculated. Abbreviations: IQR = Interquartile range; N = numerosity; UPC = Urine protein-creatinine ratio; BP = blood pressure; ALP = alkaline phosphatase; ALT = alanine aminotransferase; γGT = gamma-glutamyl transferase; CHOL = cholesterol; ALB = albumin; LDDST8= T8 cortisol concentration at the low-dose dexamethasone suppression test; Post-ACTH cortisol = post-ACTH administration cortisol concentration.

**Supplementary Table 2: Comparison between “normalizers” and “non-normalizers” to trilostane based on variables (urine protein-creatinine ratio, clinical disease score, blood pressure, trilostane daily dosage) at the last available follow-up through Wilcoxon rank-sum test.** In the “N” column is reported the total number of dogs on which the data are calculated. Abbreviations: IQR = Interquartile range; N = numerosity; UPC = Urine protein-creatinine ratio; BP = non-Blood pressure.

**Supplementary Table 3:**
